# The changing trend of teleconsultations during COVID-19 era at a tertiary facility in Tanzania

**DOI:** 10.11604/pamj.supp.2020.35.2.24977

**Published:** 2020-07-28

**Authors:** Philip Babatunde Adebayo, Ahmed Jusabani, Murtaza Mukhtar, Ali Akbar Zehri

**Affiliations:** 1 Neurology Unit, Department of Medicine, Aga Khan, University, Dar Es Salaam, Tanzania,; 2 Department of Radiology, Aga Khan Hospital, Dar es Salaam,; 3 Outpatient Service Department, Aga Khan Hospital, Dar es Salaam,; 4 Department of Surgery, Aga Khan University, Dar es Salaam

**Keywords:** COVID-19, teleconsultation, telemedicine, health services, Tanzania

## Abstract

**Introduction:**

the current COVID-19 pandemic has occasioned the increased adoption of telemedicine. This study reports the uptake and trend of a new teleconsultation service in a Tanzanian hospital.

**Methods:**

this is a retrospective observational study that profiled requests for teleconsultations and uptake of the service between April 1, 2020, and June 30, 2020.

**Results:**

two hundred and eighteen telephone inquiries were received over the 3 months. One hundred and sixteen (53.2%) individuals followed through with the teleconsultations. Paediatric (38.8%) and Internal medicine (32.8%) were the subspecialties with the highest number of teleconsultations. In a frame of 3 months, teleconsultation uptake was highest in May and lowest in June.

**Conclusion:**

there was a steady rise and a rapid fall in requests and uptake of teleconsultation services over the period under evaluation. Lack of insurance coverage for teleconsultations was a significant barrier. We propose a re-education and reiteration of the benefits of telemedicine to all stakeholders. This is important for the current era and beyond.

## Introduction

Coronavirus disease 2019 (COVID-19) caused by the SARS-CoV-2 virus is now established and spreading in most African countries following the initial spread in China, Europe, and America. The effect of the pandemic on most facets of life including healthcare delivery has been far-reaching and profound. Healthcare services have become progressively difficult to sustain because of the widespread enforcement of lockdown measures to prevent viral transmission. It had become quite difficult to attend to outpatient and non-emergency cases. The prompt attention to some emergency cases have also been affected. Furthermore, the vulnerability of the elderly population and those with underlying chronic medical conditions to COVID-19 gave rise to additional disincentives to seeking healthcare services by this category of patients even when the need was obvious and urgent. This outlook gave rise to the adoption of telemedicine across many institutions worldwide including those in Africa [1]. Tele-health or telemedicine refers to the exchange of medical information from one site to another through electronic communication to improve a patient´s health. This often employs communication technology modalities including synchronous discussion over a telephone or exchange of information through video or image [2]. Since the first case of COVID-19 in Tanzania was established on the 16th March 2020 [3] many health facilities have resorted to teleconsultations for continued patient care. The management of Aga Khan Hospital Dar es Salaam equally set up a teleconsultation platform to continue to provide outpatient services for patients who needed them. This study was aimed to investigate the trend of teleconsultation services over three months between April 1, 2020, to June 30, 2020.

## Methods

A social media (Facebook and Instagram) campaign was conducted in the first week of April (April 1-7, 2020) to sensitize the public to the availability of telemedicine as a healthcare service option during the era of COVID-19. We performed an audit of all the teleconsultations and phone inquiries by patients or their relatives during the period being evaluated (April 1, 2020, to June 30, 2020). The data was obtained from the outpatient booking department and, the information technology department of Aga Khan hospital Dar es Salaam. We collated the number of inquiries and teleconsultations in the outpatient clinics during the period. Data were computed, and descriptive analysis was performed using Microsoft Excel software. The proportion of consultations per clinic was calculated. We also generated a trend of the number of calls and teleconsultations during the period. The hospital scientific committee approved the study and the ethics and research committee of Aga University, Dar es Salaam approved the study.

## Results

**Figure 1:**
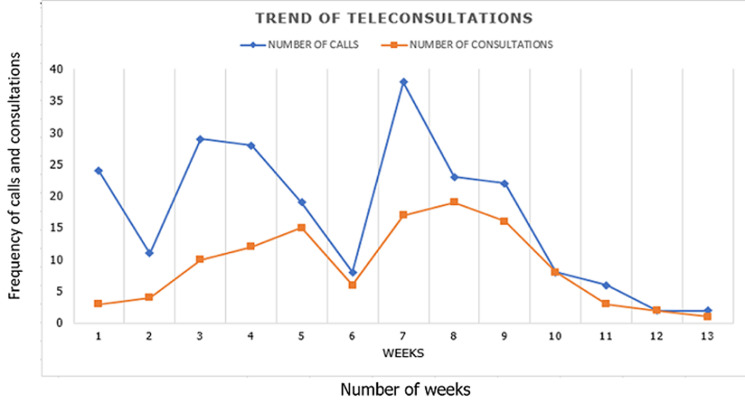
thirteen weeks trend of teleconsultations

Two hundred and eighteen telephone inquiries were received over the 3 months. One hundred and sixteen (53.2%) individuals followed through with the teleconsultations and were attended. Lack of insurance approval for teleconsultation was the reason for not booking a teleconsultation in 34 (33.3%) of the 102 individuals who did not book appointments for teleconsultation (Table 1). Table 2 shows the teleconsultations according to the specialties. Paediatric and internal medicine specialties got the highest number of teleconsultations throughout the period. Figure 1 shows the trend of the inquiries and teleconsultations over the three months under survey. Teleconsultations was highest in May 2020 and lowest in June. The trend chart shows a sharp drop in requests for teleconsultations services in June.

**Table 1:** inquiry and completion of teleconsultations

Months	Calls Requested, n (%)	Completed Teleconsultations, n (%)	Clients unable to use Insurance, n (%)
April,2020	109 (50)	44 (37.9)	21 (61.8)
May,2020	99 (45.4)	66 (56.9)	13 (38.2)
June,2020	10 (4.6)	6 (5.1)	0 (0)
Total	218 (100)	116 (100)	34 (100)

**Table 2:** teleconsultations according to specialties

Specialty	April, 2020, n (%)	May 2020, n (%)	June, 2020 n (%)
Paediatric	27 (61.4)	16 (24.2)	2 (33.3)
Internal Medicine	11 (25.0)	26 (39.4)	1 (16.6)
Family Medicine	4 (9.0)	3 (4.5)	-
Orthopaedic	1 (2.2)	2 (3.0)	-
Urology	2 (4.5)	5 (7.6)	-
Cardiology	1 (2.2)	2 (3.0)	-
Neurology	1 (2.2)	1 (1.5)	-
Dietician	-	3 (4.5)	-
OBGYN	-	4 (6.0)	2 (33.3)
Gen. Surgery	-	2 (3.0)	-
RMO	3 (6.8)	2 (3.0)	1 (16.6)
Total	44 (100)	66 (100)	6 (100)

RMO, Resident Medical Officer; OBGNY, Obstetrics and Gynecology

## Discussion

Although reserved as a tool to delivering healthcare to underserved communities especially in the rural areas in Africa, the current need to maintain physical distancing and limit viral transmission has seen an increase in the use of telemedicine; in particular, teleconsultation. Experiences shared by Song et al. suggests that telemedicine activities prevent close contact and decrease the chances of transmission of latent COVID-19 infection [4]. Health institutions in Africa are rolling out telehealth initiatives [1] while regulatory bodies are modifying protocols for telehealth [5]. Our data revealed that more than half of the patients who showed interest in teleconsultations eventually got the service. Among those who could not have teleconsultation, about one-third said the insurance companies would not pay for the consultation. Those who eventually got the service had to pay cash. Our data did not explore other barriers to having a teleconsultation among the clients although extant literature shows that lack of awareness of telemedicine options on the part of the patients; unwillingness to explore other modalities of consultations and reluctance to see a new physician are barriers to teleconsultations [6]. Our initial social media campaign was an attempt to overcome the barrier of awareness. Our patients were made aware that they could see any doctor of their choice including their regular physician.

The present data suggest that the initial enthusiasm has waned significantly in the four weeks of June. The reasons for this outlook need to be investigated albeit, we could posit that the general public perception of a reduction in COVID-19 cases could be responsible. In the immediate, to prevent waves of COVID-19 infection, the adoption of steps to encouraging teleconsultations and overcoming the barrier should be reiterated to all the stakeholders-patients, healthcare institutions and insurance companies. According to Portnoy et al [7], providing education to people that telemedicine is an effective alternative and safer under the current circumstances is important. There is also the need to institute and expand the system of reimbursement coverage for physicians who see patients through telemedicine. The main steps can be followed up with making people aware that a telemedicine benefit exists, with step-by-step instructions on how it can be accessed, while helping them to understand how telemedicine works. Efforts to reduce cost barriers to accessing telemedicine should be a continuous one [7]. While recent clinical practice in high-income countries (HIC) has witnessed a rapid incorporation of telemedicine into conventional practice [8,9], this cannot be said of sub-Saharan Africa health service delivery. Perhaps the capability of telehealth in SSA will be expanded in the immediate future, the uncertain trajectory of the current pandemic calls for an exploration of every avenue to stem the tide. Increasing research into the barriers affecting uptake of services as well as other access, financial and infrastructural issues are needed for the current era and beyond.

## Conclusion

Before the Covid-19 pandemic, teleconsultation has been an essential tool in health service delivery with a clear shift in moving healthcare from the doctor´s office to where the patient is. Teleconsultation emerged to be a highly valuable tool during the pandemic and should be considered to part of the new normal beyond the pandemic. While our data suggests a current drop in teleconsultation uptake, efforts at re-educating the public, encouraging insurance companies to reimburse physicians and policymakers to enact telemedicine policies in Africa are advocated.

### What is known about this topic

- Telemedicine is generally reserved for the geographically underserved communities;
- Telemedicine has become necessary during the COVID-19 era to encourage social distancing and prevent viral transmission;
- The use of telemedicine is increasing worldwide during COVID-era.

### What this study adds

- The uptake rate of telemedicine during COVID-era in a sub-Saharan African facility;
- The changing trends of telemedicine uptake;
- The need to revise insurance policies to include teleconsultation services in sub-Saharan Africa.
